# Supplementary material for: A Multicenter Study about the Population Treated in the Respiratory Triage Stations Deployed by the Red Cross during the COVID-19 Pandemic
Source: Int J Environ Res Public Health. 2022 Dec 25;20(1):313. doi: 10.3390/ijerph20010313 (PMC9819537; doi:10.3390/ijerph20010313)
Supplement: Supplementary file 1 [file ijerph-20-00313-s001.zip › Supplementary 1.pdf]

Table S1. Triage teams participating in the study.

PROVINCE	CANTON	TRIAGE TEAM CENTRE
Province of Azuay	Cuenca	Vicente Corral Moscoso Hospital
	Cuenca	Mariano Estrella Hospital
Province of El Oro	Machala	Velasco Ibarra Health Centre
Province of Guayas	Salitre	Salitre Health Centre
	Guayaquil	Hospital of the Bicentenary
Province of Los Ríos	Babahoyo	Enrique Ponce Maternal-Child Health Centre
	Urbaneta	Juan Montalván Hospital
Province of Manabí	Rocafuerte	Rocafuerte Health Centre
	San Vicente	San Vicente Health Centre
	Tosagua	Tosagua Health Centre
Province of Pichincha	Quito	Town's Committee Health Centre
	Quito	Cotocollao Health Centre
	Quito	Guamaní Health Centre
	Quito	San Antonio Health Centre
	Quito	Las Casas Health Centre
	Machachi	Machachi Primary-level Hospital
Province of Santo Domingo	Santo Domingo	Augusto Egas Health Centre
	Santo Domingo	Los Rosales Health Centre
	La Concordia	La Concordia Health Centre
Province of Tungurahua	Ambato	Health Centre 1-Ingahurco
	Ambato	Health Centre 2-Simón Bolívar
	Ambato	Pelileo Primary-level Hospital
