# Supplementary material for: A Multicenter Study about the Population Treated in the Respiratory Triage Stations Deployed by the Red Cross during the COVID-19 Pandemic
Source: Int J Environ Res Public Health. 2022 Dec 25;20(1):313. doi: 10.3390/ijerph20010313 (PMC9819537; doi:10.3390/ijerph20010313)
Supplement: Supplementary file 1 [file ijerph-20-00313-s001.zip › Supplementary 3.pdf]

Table S2. Descriptive analysis of the clinical and care variables.

Gender	n	%	95% CI
Male	9,107	43.12%	[43.4-43.8]
Female	12,013	56.88%	[56.2-57.5]
**Age**	34.1 years old (SD=20.02) [0-98 years old]		
0-5 years old	2,212	10.5%	[10.0-10.9]
6-12 years old	1,178	5.6%	[5.2-5.9]
13-17 years old	891	4.2%	[3.9-4.5]
18-29 years old	4,762	22.5%	[21.9-23.1]
30-39 years old	3,929	18.6%	[18.1-19.1]
40-49 years old	3,347	15.8%	[15.3-16.3]
50-59 years old	2,371	11.2%	[10.8-11.6]
60-69 years old	1,453	6.8%	[6.5-7.2]
70-79 years old	709	3.3%	[3.1-3.6]
80-89 years old	222	1.0%	[0.9-1.2]
90+ years old	42	0.2%	[0.1-0.2]
**Country of origin**	**n**	**%**	**95% CI**
Ecuador	20,630	97.68%	[97.4-97.8]
Venezuela	331	1.57%	[1.4-1.7]
Colombia	67	0.32%	[0.25-0.4]
Guatemala	42	0.20%	[0.15-0.27]
Cuba	13	0.06%	[0.04-0.11]
Peru	10	0.05%	[0.03-0.09]
Argentina	2	0.01%	[0.0-0.03]
Brazil	1	0.00%	[0.0-0.03]
Bolivia	1	0.00%	[0.0-0.03]

Chile	1	0.00%	[0.0-0.03]
Haiti	1	0.00%	[0.0-0.03]
Honduras	1	0.00%	[0.0-0.03]
Mexico	1	0.00%	[0.0-0.03]
Other countries	19	0.09%	[0.06-0.14]
**Disability**	**n**	**%**	**95% CI**
None	20,945	99.17%	[99.0-99.3]
Intellectual	52	0.25%	[0.19-0.3]
Physical	47	0.22%	[0.17-0.3]
Visual	26	0.12%	[0.08-0.18]
Auditory	19	0.09%	[0.06-0.14]
Psychosocial	16	0.08%	[0.05-0.12]
Multiple	9	0.04%	[0.02-0.08]
Language	6	0.03%	[0.01-0.06]
**CENTRE**	**n**	**%**	**95% CI**
Vicente Corral Moscoso Hospital	269	1.2%	[1.1-1.4]
Mariano Estrella Hospital	326	1.5%	[1.2-1.7]
PROVINCE OF AZUAY	**595**	**2.8%**	**[2.6-3.0]**
Velasco Ibarra Health Centre	1,929	9.1%	[8.7-9.5]
PROVINCE OF EL ORO	1,929	9.1%	[8.7-9.5]
Salitre Health Centre	1,856	8.8%	[8.4-9.1]
Hospital of the Bicentenary	2,671	12.6%	[12.2-13.1]
PROVINCE OF GUAYAS	4,527	21.4%	[20.9-22.0]
Enrique Ponce Maternal-Child Health Centre	673	3.2%	[2.9-3.4]
Juan Montalván Hospital	792	3.7%	[3.5-4.0]
PROVINCE OF LOS RÍOS	1,465	6.9%	[6.6-7.3]

Rocafuerte Health Centre	1,001	4.7%	[4.4-5.0]
San Vicente Health Centre	839	3.9%	[3.7-4.2]
Tosagua Health Centre	414	1.9%	[1.7-2.1]
PROVINCE OF MANABÍ	2,254	10.6%	[10.2-11.1]
Town's Committee Health Centre	686	3.5%	[3.0-3.5]
Cotocollao Health Centre	628	2.9%	[2.7-3.2]
Guamaní Health Centre	949	4.5%	[4.2-4.8]
San Antonio Health Centre	551	2.6%	[2.4-2.8]
Las Casas Health Centre	508	2.4%	[2.2-2.6]
Machachi Primary-level Hospital	345	1.6%	[1.4-1.8]
PROVINCE OF PICHINCHA	3,667	17.3%	[16.8-17.8]
Augusto Egas Health Centre	1,048	4.9%	[4.7-5.2]
Los Rosales Health Centre	1,398	6.6%	[6.3-6.9]
La Concordia Health Centre	743	3.5%	[3.2-3.7]
PROVINCE SANTO DOMINGO	3,189	15.1%	[14.6-15.6]
Health Centre 1-Ingahurco	1,392	6.6%	[6.2-6.9]
Health Centre 2-Simón Bolívar	813	3.8%	[3.6-4.1]
Pelileo Primary-level Hospital	1,289	6.1%	[5.8-6.4]
PROVINCE OF TUNGURAHUA	3,494	16.5%	[16.0-17.0]
TOTAL	21,120	100%	
