# Supplementary figures and images for: A Multicenter Study about the Population Treated in the Respiratory Triage Stations Deployed by the Red Cross during the COVID-19 Pandemic
Source: Int J Environ Res Public Health. 2022 Dec 25;20(1):313. doi: 10.3390/ijerph20010313 (PMC9819537; doi:10.3390/ijerph20010313)

**Figure S1.** Care path for COVID-19.

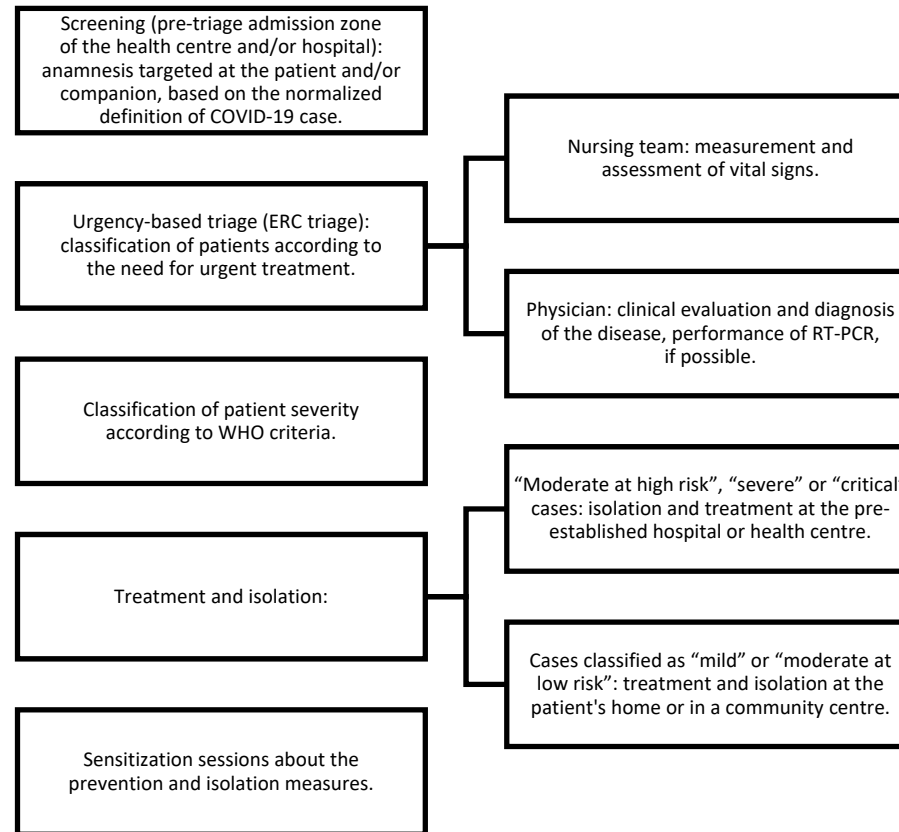

Supplement: Supplementary file 1 [file ijerph-20-00313-s001.zip › Supplementary 2.pdf]
